# Prevalence and factors associated with depression and anxiety among COVID-19 survivors in Dhaka city

**DOI:** 10.3389/fpsyt.2024.1280245

**Published:** 2024-01-23

**Authors:** Md. Golam Kibria, Russell Kabir, Ummay Salma Rahman, Shakil Ahmed, SM Sayadat Amin, Md. Mahbubur Rahman, SM Yasir Arafat

**Affiliations:** ^1^ Department of Research, Centre for Development Action, Dhaka, Bangladesh; ^2^ School of Allied Health, Anglia Ruskin University, Bishop Hall Lane, Chelmsford, United Kingdom; ^3^ Upstream Lab, St. Michael’s Hospital, Toronto, Ontario, ON, Canada; ^4^ Maternal and Child Health Division, International Centre for Diarrhoeal Disease Research, Bangladesh, Dhaka, Bangladesh; ^5^ Department of Research and Publication, Bangladesh Medical Research Council, Dhaka, Bangladesh; ^6^ Department of Health, Noora Health, Dhaka, Bangladesh; ^7^ Department of Psychiatry, Enam Medical College, Dhaka, Bangladesh

**Keywords:** depression, anxiety, prevalence, COVID-19 survivors, Dhaka city

## Abstract

**Background:**

Coronavirus disease 2019 (COVID-19) is a global public health concern. Evidence shows that depression and anxiety are common among patients with COVID-19 after recovery. About one-third of the total COVID-19 cases in Bangladesh have been reported in Dhaka city. Therefore, the study aimed to evaluate the prevalence of depression and anxiety among COVID-19 survivors in Dhaka city as well as to identify the factors associated with these mental health conditions.

**Methods:**

A cross-sectional study was carried out among a total of 384 COVID-19 survivors aged 18 years or older. Data collection was done through face-to-face and telephone interviews using a semi-structured questionnaire. Patient Health Questionnaire (PHQ-9) and Generalized Anxiety Disorder (GAD-7) scales were used to assess depression and anxiety, respectively. Binary logistic regression analysis was performed to identify the predictors of depression and anxiety among patients recovered from COVID-19.

**Results:**

The overall prevalence of depression and anxiety was 26.0% and 23.2%, respectively among COVID-19 survivors. The respondents who were ≥60 years were 2.62 and 3.02 times more likely to report depressive and anxiety symptoms, respectively than those aged 18 to 39 years. Hospitalised patients recovered from COVID-19 had a 2.18 times higher chance of developing anxiety than their non-hospitalised counterparts. COVID-19 recovered patients with comorbidities were at 3.35 and 2.97 times higher risk of depression and anxiety, respectively compared to those without comorbidities. Similarly, the respondents who had already passed a period of 15 days to 3 months after recovery showed 3.06 and 1.85 times higher odds of depression and anxiety, respectively than those who had already passed a period of above 3 to 6 months after recovery.

**Conclusion:**

The study reported a high prevalence of depression and anxiety among COVID-19 survivors living in Dhaka city. The findings suggest the need for appropriate interventions to reduce mental health complications in COVID-19 survivors.

## 1 Introduction

The COVID-19 pandemic has emerged as a significant global public health issue. The etiology of this communicable ailment can be attributed to the pathogen known as severe acute respiratory syndrome coronavirus 2 (SARS-CoV-2) (1). Since its initial identification in Wuhan city, China in early December 2019, the COVID-19 pandemic has resulted in a global death toll over 6 million people as of 19 November 2023 (2). The very first occurrence of COVID-19 was detected in Dhaka, the capital city of Bangladesh, on 8 March 2020 (3). As of 27 March 2022, Dhaka city has emerged as the primary location for COVID-19 cases and fatalities in Bangladesh, constituting 30.2% of the nation’s overall COVID-19 cases and and 10.3% of COVID-19 deaths (4). This city is recognised as one of the world’s most densely populated cities, exhibiting a population density of 30,093 residents per square kilometer (5). The elevated population density has rendered the residents of Dhaka city more susceptible to COVID-19 infection in comparison to those of other cities within the country (6).

The 21^st^ century has witnessed the emergence of three notable epidemics of viral respiratory diseases, namely severe acute respiratory syndrome (SARS), Middle East respiratory syndrome (MERS), and COVID-19. These epidemics were caused by coronaviruses (CoVs) (7). Because of its higher reproduction rate, SARS-CoV-2 has infected more people globally compared to other epidemics, such as SARS and MERS (8, 9). Ample evidence suggests that people who have survived the COVID-19 pandemic have a heightened susceptibility to various mental health issues, of which depression and anxiety are more common (10–12). There are greater variations in the global prevalence of depression and anxiety among COVID-19 survivors. Interestingly, 10.8% to 61.3% of individuals who recovered from COVID-19 reported experiencing symptoms of depression, while percentages ranging from 13.5% to 44.5% were observed in relation to the development of anxiety (10, 13–15).

Depression and anxiety impose significant detrimental impacts on the overall well-being of individuals. Existing research indicated a correlation between depression and the occurrence of cardiovascular illnesses, stroke, and hypertension (16–18). As reported by a research study, those diagnosed with depression exhibited a significantly elevated likelihood of engaging in suicide attempts, with a risk around 11 times greater compared to those who did not manifest the disease (19). It has been identified as the largest contributor of disability (20). A separate research investigation examined the impact of depression on job productivity, revealing that those experiencing depression showed a reduction of 5.6 hours in their weekly productive time at their workplace, in contrast to their non-depressed peers who had a decrease of 1.6 hours (21). Another previous study reported that those experiencing anxiety exhibited a heightened susceptibility to cardiovascular diseases and hypertension (22). Anxiety ranks as the sixth leading cause of global disability (23) and has shown a significant correlation with suicide attempts (24). Moreover, individuals diagnosed with anxiety disorders exhibited a 1.4-fold increased likelihood of experiencing diminished work performance compared to those without such conditions (25).

Approximately one-third of the nation’s aggregate COVID-19 cases are documented within the radius of Dhaka city. Drawing upon earlier global findings on post-COVID-19 psychological sequelae, s we hypothesised that a significant proportion of individuals who recovered from COVID-19 in Dhaka city would manifest symptoms of depression and anxiety. In Bangladesh, there have been numerous studies to investigate the mental health status of people during the coronvirus outbreak (26–29). To date, little research has been conducted in the country to evaluate psychological outcomes in survivors of COVID-19 (30, 31). This indicates that there is still no adequate information to estimate the burden of mental illness in recovered patients in the capital. Against this backdrop, we conducted this study to evaluate the prevalence of depression and anxiety among individuals who recovered from COVID-19 in Dhaka city and to identify any factors that might be connected with these mental health conditions. The findings of this study can be used to strengthen the provision of mental healthcare services, especially in the capital to address the mental health needs of COVID-19 survivors during and after the pandemic.

## 2 Methods

### 2.1 Study design and setting

We conducted a cross-sectional study in Dhaka city, Bangladesh. The study was carried out from 19 January to 30 June 2021.

### 2.2 Study participants

We collected data from a sample of hospitalised and non-hospitalised COVID-19 survivors residing in Dhaka city. The respondents of this study were included under the following criteria – (a) adults aged 18 years or older and (b) those who tested negative for COVID-19 over a period of 15 days to 6 months (1 November 2020 to 15 April 2021) during data collection.The study excluded COVID-19 survivors with speech and/or hearing impairments to avoid communication barriers.We included both hospitalised and non-hospitalised patients to determine whether there was any difference in the prevalence rate of depression and anxiety between the groups. Hospitalised patients were recruited from one government COVID-19 dedicated hospital and one private hospital with a COVID-19 unit situtated in Dhaka city.

### 2.3 Sampling strategy

The minimum sample size for this study was calculated at 384 using a single population proportion formula,n = $\frac{Z^{2}pq}{e^{2}}$ (32), where n = desired sample size, z = standard normal deviate = 1.96 at 95% confidence interval (CI), p = prevalence estimate = 0.5 as there was no reported prevalence of depression and anxiety among COVID-19 survivors in Dhaka city at the time when the present study was designed, q=1- p, and e = precision of the prevalence estimate = 0.05.

We allocated the sample of 384 to hospitalised and non-hospitalised patients equally; thus, each group had a sub-sample of 192. Because of administrative difficulties and confidentiality issues, we were able to collect the medical records of only 500 COVID-19 patients from the two hospitals. Out of these 500 patients, 192 were randomly selected, and on the other hand, another 192 non-hospitalised patients were selected using the snowball sampling technique.

### 2.4 Measures

#### 2.4.1 Basic characteristics

The study collected information on the respondents’ basic characteristics: sex (male or female), age in full years, marital status (unmarried or ever married), education level (none/primary, seconday, higher secondary, or bachelor’s degree and above), occupational category (service holder, housewife, retired person, businessman, health professional, or student), religion (Muslim, Hindu, or Buddhist), monthly family income, hospitalisation status (hospitalised or non-hospitalised), comorbidity (present or absent), and time after recovery. Health professionals included medical doctors, nurses, physiotherapists, and other hospital staff. We asked the respondents about their monthly family income and then categorised the income into three levels: <50,000 BDT, 50,000 to 99,000 BDT, and ≥100,000 BDT. Similarly, we collected data on the number of days the respondents had already passed after recovery from COVID-19, and in the analysis stage, we classified the time into two categories: 15 days to 3 months and above 3 to 6 months.

#### 2.4.2 Patient health questionnaire (PHQ-9)

The Patient Health Questionnaire (PHQ-9) scale is a widely used instrument to assess the presence and levels of depressive symptoms in an individual over the past two weeks. This scale consists of 9 items regarding depressive symptoms with a four-point Likert scale (0 = not at all, 1 = several days, 2 = more than half of the days, and 3 = nearly every day) (33). The levels of depression were categorised as minimal/no, mild, moderate, moderately severe and severe based on scoring in the range of 0- 4,5- 9,10- 14,15- 19, and 20- 27, respectively (34). Later, a cutoff score of ≥5 was used to indicate the presence of depression, whereas a cutoff score of ≤4 to indicate the absence of depression (35). The validated Bangla version PHQ-9 scale was used in the present study (36). However, in this study, the PHQ-9 scale showed good reliability (Cronbach’s alpha = 0.855 and mean inter-item correlation = 0.420).

#### 2.4.3 Generalized anxiety disorder (GAD-7)

The Generalized Anxiety Disorder (GAD-7) scale is a standard instrument to assess the presence and levels of anxiety in an individual over the past two weeks. This scale consists of 7 items having a four-point Likert scale (0 = not at all, 1 = several days, 2 = more than half of the days, and 3 = nearly every day) (37). The levels of anxiety were categorised as minimal/no at a total score of 0- 4, mild at 5- 9, moderate at 10- 14 and severe at 15- 21 (38), and then we used a cutoff score of ≥5 to indicate the presence of anxiety and a cutoff score of ≤4 to indicate the absence of anxiety (39). In this study, the Bangla version GAD-7 scale (40) was used to assess the presence and levels of anxiety in COVID-19 survivors. In our study, the GAD-7 scale was found to have good reliability (Cronbach’s alpha = 0.857 and mean inter-item correlation = 0.471).

### 2.5 Training and data collection

For this study, we recruited five medical science and social science graduates and trained them for two days on the objectives and methodology of the study and the survey questionnaire before data collection. Data collectors communicated with each of the selected patients over the phone and fixed the interview time at least 3 days before data collection. On the scheduled dates, data collectors met the respondents in their houses or offices and interviewed them using a semi-structured survey questionnaire after obtaining written consent from them. Some respondents were unwilling to participate in the face-to-face interviews for fear of contacting COVID-19, and this led us to conduct telephone interviews. Finally, we completed a total of 384 interviews, including 282 in-person and 102 telephone interviews from 1 May to 10 May 2021. Each interview lasted for approximately 20 minutes. Safety measures, such as physical distancing and face mask use were strictly maintained during the time of face-to-face interviews. However, the respondents were not given financial benefits for participating in the study.

### 2.6 Statistical analysis

Data were analysed using SPSS statistical software version 25. Descriptive statistics (frequencies and percentages) were used to describe the characteristics of the sample. To determine the associations between categorical variables, the chi-square test of independence was used. If any of the categorical variables showed expected frequency less than 5 observations in any of the cells, Fisher’s exact test was performed. Binary logistic regression was conducted to compute adjusted odds ratio (AOR) with 95% confidence intervals (CIs) to identify the associated factors of depression and anxiety among COVID-19 survivors. Only those independent variables that were found to be statistically significant *(p*-value<0.05) in bivariate analyses were entered into the the binary logistic regression (LR) models, and these variables were age, education level, occupational category, monthly family income, hospitalisation status, comorbidity, and time after recovery. The Hosmer-Lemeshow test was used to assess the goodness of fit of the LR models. The significance values for the models predicting depression (M1) and anxiety (M2) were 0.994 and 0.975, respectively, which are greater than p<0.05, indicating that these models fit the data. We checked for multicollinearity in the LR models by observing the standard errors (SEs) of regression coefficients in the logistic regression analyses. An SE >5.0 indicates the presence of multicollinearity between independent variables (41). In this study, the SEs of regression coefficients in the models M1 and M2 were 0.116 and 0.121, respectively, and therefore there was no multicollinearity between the independent variables. All statistics were tested using a two-sided test, and a *p*-value of <0.05 was considered statistically significant.

### 2.7 Ethical statement

This study involving human subjects was reviewed and approved by the National Research Ethics Committee of the Bangladesh Medical Research Council (BMRC) (Ref: BMRC/HPNSP-Research Grantl2020-2021 I 52(1 -47). The patients/participants provided either written or verbal informed consent to participate in this study.

## 3 Results

### 3.1 Basic chracteristics and prevalence of depression and anxiety

A total of 384 respondents were included in this study. **Table 1** illustrates the bivariate analysis results of COVID-19 survivors’ depression and anxiety with their basic characteristics. Most of the respondents were males (57.8%), young adults aged 18 to39 years (43.0%), ever married (81.8%), at least graduates (78.9%), service holders (41.1%), Muslims (92.7%), and had a monthly family income of BDT<50,000 (50.3%). Half of the respondents were hospitalised, and the other half were not hospitalised when they were COVID-19 positive. Among all the respondents, 37.5% were found to have at least one noncommunicable disease. More than half of the respondents (54.4%) had already passed a period of 15 days to 3 months after recovery from COVID-19, while 45.6% had already passed a period of above 3 to 6 months after recovery from COVID-19.

**Table 1:** Distribution of variables and their association with depression and anxiety among COVID-19 survivors.

Variable	Total =384		Depression				Anxiety			
			Present				Present			
	n	%	n	(%)	χ2^†^	*p*-value	n	%	χ2^†^	*p*-value
**Overall**			100	26.0			89	23.2		
Sex										
Male	222	57.8	51	23.0	2.573	0.109	47	21.2	1.189	0.275
Female	162	42.2	49	30.2			42	25.9		
Age										
18 to 39 years	165	43.0	23	13.9	48.463	**<0.001**	19	11.5	60.754	**<0.001**
40 to 59 years	137	35.7	32	23.4			25	18.2		
≥ 60 years	82	21.4	45	54.9			45	54.9		
Marital status										
Unmarried	70	18.2	13	18.6	2.480	0.115	11	15.7	2.678	0.102
Ever married	314	81.8	87	27.7			78	24.8		
Education level										
None/Primary	16	4.2	9	56.3	10.273	**0.016**	10	62.5	18.045	**<0.001**
Secondary	19	4.9	6	31.6			7	36.8		
Higher secondary	46	12.0	15	32.6			12	26.1		
Bachelor’s degree and above	303	78.9	70	23.1			60	19.8		
Occupational category										
Service holder	158	41.1	27	17.1		**<0.001** ^F^	22	13.9		**<0.001^F^**
Housewife	86	22.4	29	33.7			26	30.2		
Retired person	45	11.7	23	51.1			23	51.1		
Businessman	32	8.3	9	28.1			8	25.0		
Health professional	32	8.3	8	25.0			6	18.8		
Student	31	8.1	4	12.9			4	12.9		
Religion										
Muslim	356	92.7	95	26.7		0.480^F^	83	23.3		0.752^F^
Hindu	25	6.5	4	16.0			5	20.0		
Buddhist	3	0.8	1	33.3			1	33.3		
Monthly family income										
< 50,000 BDT	193	50.3	60	31.1	7.496	**0.024**	60	31.1	13.706	**0.001**
50,000 - 99,000 BDT	127	33.1	31	24.4			20	15.7		
≥100,000 BDT	64	16.7	9	14.1			9	14.1		
Hospitalisation status										
Hospitalised	192	50.0	71	37.0	23.851	**<0.001**	66	34.4	27.043	**<0.001**
Non-hospitalised	192	50.0	29	15.1			23	12.0		
Comorbidity										
Present	144	37.5	66	45.8	46.859	**<0.001**	60	41.7	44.237	**<0.001**
Absent	240	62.5	34	14.2			29	12.1		
Time after recovery										
15 days to 3 months	209	54.4	69	33.0	11.577	**0.001**	57	27.3	4.321	**0.038**
Above 3 to 6 months	175	45.6	31	17.7			32	18.3		

^†^Pearson’s chi-square; ^F^ *p*-value based on Fisher’s exact test.

Bold values indicate statistically significant result.

The overall prevalence of depression among the respondents was 26.0%. Depression was found to be more prevalent in individuals aged ≥60 years (54.9%, p<0.001), those respondents who had no or primary education (56.3%, p=0.016), retired persons (51.1%, p<0.001), those whose families earned<50,000 BDT per month (31.1%, p=0.024), hospitalised patients (37.0%, p<0.001), patients with comorbidities (45.8%, p<0.001), and those who had already passed a period of 15 days to 3 months after recovery (33.0%, p=0.001).

In this study, the overall prevalence of anxiety was 23.2%. Anxiety was higher in COVID-19 survivors aged ≥60 years (54.9%, p<0.001), those having no or primary education (62.5%, p<0.001), retired persons (51.1%, p<0.001), those respondents whose monthly family income was<50,000 BDT (31.1%, p=0.001), hospitalised patients (34.4%, p<0.001), comorbid respondents (41.7%, p<0.001), and patients who had already passed a period of 15 days to 3 months after recovery from COVID-19 (27.3%, p<0.038).

### 3.2 Comorbidity status of the respondents

**Figure 1:**
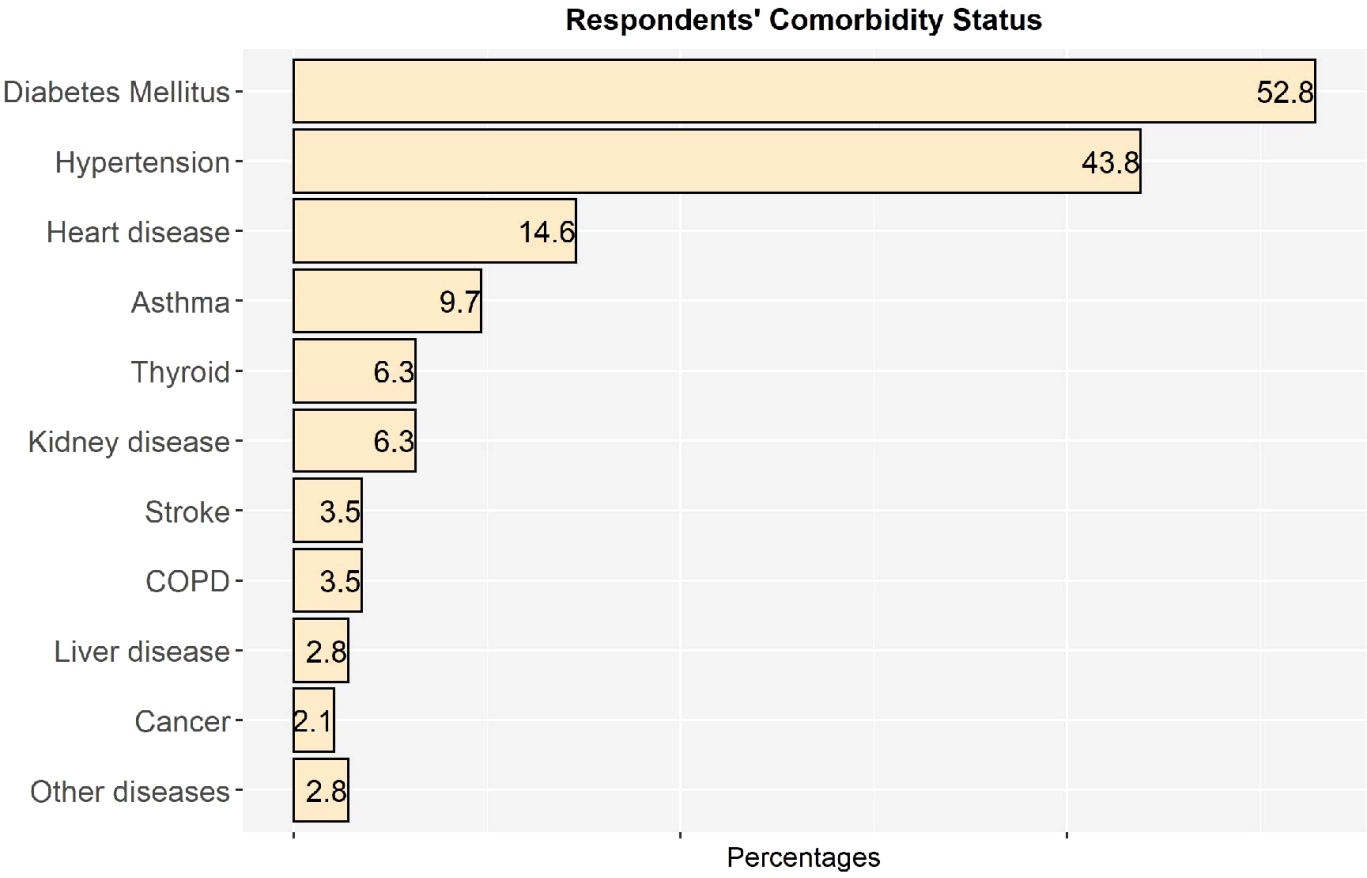
Distribution of comorbid respondents by noncommunicable diseases.

**Figure 1** illustrates the comorbidity status of the respondents,which was determined based on the results of multiple response analysis. Among 144 comorbid respondents, 52.8% had diabetes mellitus, followed by hypertension (43.8%), heart disease (14.6%), asthma (9.7%), kidney disease (6.3%), thyroid (6.3%), stroke (3.5%), chronic obstructive pulmonary disease (COPD) (3.5%), liver disease (2.8%), cancer (1.9%), and other diseases (2.8%).Other diseases included chronic pain, epilepsy, gout, and paralysis.

### 3.3 Levels of depression and anxiety

**Figure 2:**
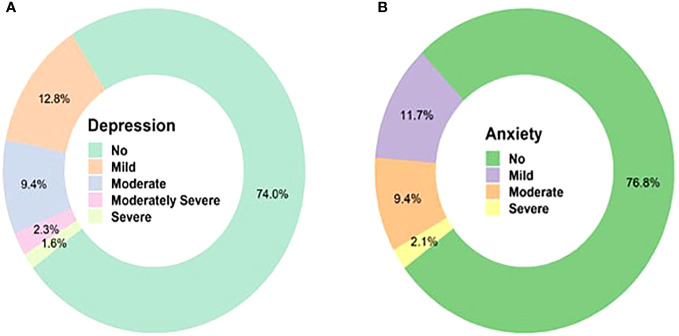
Levels of depression and anxiety among COVID-19 survivors.

**Figure 2** displays the levels of depression and anxiety that the respondents experienced after recovering from COVID-19. More than one-fourth of the respondents (26.0%) developed some sort of depressive symptoms, and among them, 12.8% had mild depression, 9.4% had moderate depression, 2.3% had moderately severe depression, and only 1.6% had severe depression. Similarly, among those who reported experiencing anxiety (23.2%),11.7% had mild anxiety, 9.4% had moderate anxiety, and 2.1% had severe anxiety.

### 3.4 Predictors of depression and anxiety

Predictors of depression and anxiety among COVID-19 survivors are presented in **Table 2**. The respondents whose age was ≥60 years were 2.62 times more likely to have depressive symptomsthan those aged 18 to 39 years (95% CI= 1.011 to 6.802). COVID-19 survivors with comorbidities were 3.35 times more likely to have depressive symptoms than those without comorbidities (95% CI= 1.810 to 6.205). Likewise, the respondents who had already passed a period of 15 days to 3 months after recovery had a 3.06 times higher chance of having depression than those who had already passed a period of above 3 to 6 months after recovery (95% CI= 1.738 to 5.379).

**Table 2:** Multivariable associations of depression and anxiety with the respondents’ basic characteristics.

Variables	Depression			Anxiety		
	AOR	95% CI	*p-*value	AOR	95% CI	*p*-value
Age						
18 to 39 years	Ref			Ref		
40 to 59 years	1.16	0.554 to 2.427	0.695	0.99	0.442 to 2.213	0.979
60+ years	2.62	1.011 to 6.802	0.047*	3.02	1.125 to 8.109	0.028*
Hospitalisation status						
No-hospitalised	Ref			Ref		
Hospitalised	1.84	0.983 to 3.434	0.056	2.18	1.115 to 4.259	0.023*
Comorbidity						
Absent	Ref			Ref		
Present	3.35	1.810 to 6.205	<0.001***	2.97	1.550 to 5.681	0.001**
Time after recovery						
Above 3 to 6 months	Ref					
15 days to 3 months	3.06	1.738 to 5.379	<0.001***	1.85	1.042 to 3.293	0.036*

Variables adjusted for in both regression models: age, education level, occupational category, monthly family income, hospitalisation status, comorbidity, and time after recovery.

AOR, Adjusted Odds Ratio; CI, Confidence Interval; *p <0.05; **p <0.01; ***p <0.001.

Bold values indicate statistically significant result.

COVID-19 survivors aged ≥60 years were at 3.02 times higher risk of developing anxiety compared to those aged 18 to 39 years (95% CI= 1.125 to 8.109). Hospitalised patients had a 2.18 times higher chance of experiencing anxiety than their non-hospitalised counterparts (95% CI= 1.115 to 4.259). The respondents who had comorbidities were 2.97 times more likely to have anxiety than those who had no comorbidities (95% CI= 1.550 to 5.681). Moreover, the respondents who had already passed a period of 15 days to 3 months after recovery from COVID-19 had a 1.85 times higher chance of having anxiety than those who had already passsed a period of above 3 to 6 months after recovery from COVID-19 (95% CI= 1.042 to 3.293).

## 4 Discussion

The present study assessed the prevalence of depression and anxiety as well as identified its associated factors in a sample of COVID-19 survivors who had already passed 15 days to 6 months after recovery. This study’s overall prevalence of depression and anxiety was 26.0% and 23.2%, respectively. These findings are comparable with those of other studies that investigated the depression and anxiety status of recovered COVID-19 patients using the PHQ-19 and GAD-7 scales. A cohort study conducted among a sample of COVID-19 survivors after discharge from a hospital in Porto, Portugal found that 29% and 23% of the respondents experienced depressive and anxiety symptoms (42). A study done in Spain showed that after discharge, 26.8% of patients with COVID-19 had depression, and 29.6% had anxiety (43). In a study in Zhongshan, China, it was observed that 29.2% of non-hospitalised COVID-19 patients had depressive symptoms, and 20.8% had anxiety (44). The slight variations that exist in the prevalence of depression and anxiety in our study and other studies compared could be due to study design, study population, study location, and hospitalisation status of participants. However, this high prevalence suggests an urgent need for psychiatric treatment and rehabilitation for COVID-19 survivors.

Age was found to be statistically significant with depression and anxiety in the present study. The findings of this study showed that older adults aged 60 years or above had a higher risk of experiencing depression and anxiety compared to younger adults aged 18 to 39 years. A study conducted among patients with COVID-19 admitted to isolation facilities in Bangladesh revealed that depression and anxiety disorders were more prevalent in the respondents who were 50 years or older (45). In a study done in northern India, it was found that COVID-19 survivors who were aged ≥50 years experienced significantly higher levels of depression and anxiety than those aged 30 to 49 years (15). Another study done in the Wuhan city of China demonstrated that the prevalence of depression and anxiety was higher in patients with COVID-19 aged above 50 years (46). The higher rates of depression and anxiety among older adults can be explained by two factors: economic loss and fear of losing family members. Many old people aged around 60 years often have the responsibility to maintain their families. The pandemic has disrupted economic activities all around, putting them at risk of economic loss. Secondly, the message that older adults are at higher risk of dying from coronavirus infections may have led to psychological problems in the old age group. However, these findings suggest the need for social support and psychological interventions for COVID-19 survivors, especially for older people.

From the findings of this study, it was observed that there was a significant association between hospitalisation status and the prevalence of anxiety. Our respondents who were hospitalised reported higher levels of anxiety symptoms compared to non-hospitalised patients. This finding is consistent with the results of other epidemiological studies that assessed the psychological consequences of COVID-19 infection on recovered patients (47, 48). The higher prevalence of psychological disorders like anxiety in hospitalised patients in our study could be attributed to isolation from family members, reduced mobility, disease severity and complications, treatment expenditure, and death of other inpatients. On the other hand, several studies illustrated that non-hospitalised COVID-19 patients were at higher risk of developing anxiety compared to their hospitalized counterparts, which contradicts our finding (49–51). Therefore, further longitudinal research is required to confirm whether anxiety is higher in hospitalized patients than in non-hospitalsed patients.

In the present study, COVID-19 survivors with comorbidities were more likely to experience depressive and anxiety symptoms than those who did not have any comorbidity. This finding is compatible with the results of other previous studies conducted among COVID-19 patients in Bangladesh (45) and Cameroon (52). Evidence suggests that patients with comorbidities require more emergency services (53, 54), but in developing countries like Bangladesh, emergency services are not easily accessible. Therefore, health providers should provide emergency treatment to high risk populations like those with comorbidities through telemedicine sessions during pandemic outbreaks. Also, it is equally important to offer timely counselling to COVID-19 survivors who have multiple comorbidities to maintain a high level of mental health.

In the present study, COVID-19 survivors who had already passed a period of 15 days to 3 months after recovery were more likely to experience depressive and anxiety symptoms than those who had already passed a period of above 3 to 6 months after recovery. These findings corroborate and extend the results of earlier studies. A cohort study conducted in Italy, involving 196 patients recovered from COVID-19 revealed that 32.1% and 34.5% of the subjects had depressive and anxiety symptoms, respectively at 4-month visit, while the percentages reduced to 19.1% and 16.9%, respectively at 12-month visit (55). It was found from a cohort study done in the Rotterdam city of the Netherlands that 29.0% of patients with COVID-19 experienced anxiety at 1 month after discharge, and 20.0% experienced anxiety at 3 months after discharge (56). On the contrary, two longitudinal cohort studies that investigated the mental health outcomes of COVID-19 survivors showed that they had higher levels of depression and anxiety at 12 months than at 6 months after discharge (57, 58). These findings indicate that there is still a lack of clarity as to whether mental health outcomes in patients recovered from COVID-19 improve or deteriorate as time after recovery increases. Therefore, more extended follow-up studies are needed to understand the severity of psychiatric problems among COVID-19 survivors at different points in time after recovery.

## 5 Limitations

This study has several limitations. First of all, due to the cross-sectional design, the study cannot establish a temporal relationship between outcome and exposure variables because both are observed at a single point in time (59). Second, in this study, we did not consider the pre-existing depressive and anxiety symptoms of the respondents. This has made us unable to distinguish between pre-existing and new symptoms. Third, we did not collect clinical data, such as disease severity, need for oxygenation, and mechanical ventilation from COVID-19 survivors. If we had included these variables in the study, they could have enriched the findings. Fourth,we selected hospitalised patients randomly and non-hospitalised patients using the snowball sampling technique. This may limit the generalization of the findings. Fifth, those who refused to participate in the study may have different characteristics from the respondents, such as hospitalisation experience, psychosocial status, and resilience. This may have contributed to selection bias. Finally, this study used the phone interview mode to collect data from a large number of respondents. The absence of body language in the phone interview may have caused poor communications between the interviewers and the respondents, affecting the results of this study.

## 6 Conclusion

The present study reported a high prevalence of depression and anxiety among COVID-19 survivors in Dhaka city. Our findings showed that female sex, older age, hospitalisation, comorbidity, and shorter duration after recovery were significantly associated with depression and anxiety. These findings suggest the need for appropriate interventions to reduce mental health complications in COVID-19 survivors.

## Data availability statement

The raw data supporting the conclusions of this article will be made available by the authors, without undue reservation.

## Ethics statement

This study involving human subjects was approved by the National Research Ethics Committee of the Bangladesh Medical Research Council. The study was conducted in accordance with the local legislation and institutional requirements. The participants provided their written informed consent to participate in this study.

## Author contributions

MK: Conceptualization, Data curation, Formal Analysis, Funding acquisition, Investigation, Methodology, Project administration, Writing – original draft. RK: Conceptualization, Formal Analysis, Writing – original draft, Writing – review & editing. UR: Conceptualization, Data curation, Writing – review & editing. SA: Conceptualization, Formal Analysis, Writing – review & editing. SAm: Conceptualization, Formal Analysis, Writing – review & editing. MR: Data curation, Writing – review & editing. SAr: Funding acquisition, Writing – review & editing.
